# Autism Spectrum Disorder and Childhood Apraxia of Speech: Early Language-Related Hallmarks across Structural MRI Study

**DOI:** 10.3390/jpm10040275

**Published:** 2020-12-12

**Authors:** Eugenia Conti, Alessandra Retico, Letizia Palumbo, Giovanna Spera, Paolo Bosco, Laura Biagi, Simona Fiori, Michela Tosetti, Paola Cipriani, Giovanni Cioni, Filippo Muratori, Anna Chilosi, Sara Calderoni

**Affiliations:** 1 IRCCS Fondazione Stella Maris, 56128 Pisa, Italy; eugenia.conti@fsm.unipi.it (E.C.); p.bosco@fsm.unipi.it (P.B.); l.biagi@fsm.unipi.it (L.B.); s.fiori@fsm.unipi.it (S.F.); m.tosetti@fsm.unipi.it (M.T.); p.cipriani@fsm.unipi.it (P.C.); g.cioni@fsm.unipi.it (G.C.); f.muratori@fsm.unipi.it (F.M.); anna.chilosi@fsm.unipi.it (A.C.); 2 National Institute for Nuclear Physics (INFN), Pisa Division, 56127 Pisa, Italy; alessandra.retico@pi.infn.it (A.R.); letizia.palumbo@pi.infn.it (L.P.); giovanna.spera@pi.infn.it (G.S.); 3 Department of Clinical and Experimental Medicine, University of Pisa, 56126 Pisa, Italy

**Keywords:** Autism Spectrum Disorders (ASD), childhood apraxia of speech, children, Magnetic Resonance Imaging (MRI), neuroanatomy, FreeSurfer

## Abstract

Autism Spectrum Disorder (ASD) and Childhood Apraxia of Speech (CAS) are developmental disorders with distinct diagnostic criteria and different epidemiology. However, a common genetic background as well as overlapping clinical features between ASD and CAS have been recently reported. To date, brain structural language-related abnormalities have been detected in both the conditions, but no study directly compared young children with ASD, CAS and typical development (TD). In the current work, we aim: (i) to test the hypothesis that ASD and CAS display neurostructural differences in comparison with TD through morphometric Magnetic Resonance Imaging (MRI)-based measures (ASD vs. TD and CAS vs. TD); (ii) to investigate early possible disease-specific brain structural patterns in the two clinical groups (ASD vs. CAS); (iii) to evaluate predictive power of machine-learning (ML) techniques in differentiating the three samples (ASD, CAS, TD). We retrospectively analyzed the T1-weighted brain MRI scans of 68 children (age range: 34–74 months) grouped into three cohorts: (1) 26 children with ASD (mean age ± standard deviation: 56 ± 11 months); (2) 24 children with CAS (57 ± 10 months); (3) 18 children with TD (55 ± 13 months). Furthermore, a ML analysis based on a linear-kernel Support Vector Machine (SVM) was performed. All but one brain structures displayed significant higher volumes in both ASD and CAS children than TD peers. Specifically, ASD alterations involved fronto-temporal regions together with basal ganglia and cerebellum, while CAS alterations are more focused and shifted to frontal regions, suggesting a possible speech-related anomalies distribution. Caudate, superior temporal and hippocampus volumes directly distinguished the two conditions in terms of greater values in ASD compared to CAS. The ML analysis identified significant differences in brain features between ASD and TD children, whereas only some trends in the ML classification capability were detected in CAS as compared to TD peers. Similarly, the MRI structural underpinnings of two clinical groups were not significantly different when evaluated with linear-kernel SVM. Our results may represent the first step towards understanding shared and specific neural substrate in ASD and CAS conditions, which subsequently may contribute to early differential diagnosis and tailoring specific early intervention.

## 1. Introduction

Autism Spectrum Disorder (ASD) and Childhood Apraxia of Speech (CAS) are developmental disorders with distinct definitions and diagnostic criteria. Specifically, ASD includes a set of neurodevelopmental disorders characterized by social communication difficulties as well as restricted interests, repetitive activities and sensory abnormalities [1]. Recent estimates report an ASD prevalence of about one in 87 children aged 7–9 years [2] in Italy. ASD is a highly heterogeneous group of disorders, with multiple genetic backgrounds that may reflect multiple neuroanatomical underpinnings, which in turn are expressed with diverse behavioral manifestations [3,4].

CAS is a neurological childhood speech motor disorder in which the precision and consistency of movements underlying speech are impaired in the absence of neuromuscular deficits [5], and is included among Speech Sound Disorders (SSD) in the Diagnostic and Statistical Manual of Mental Disorders (DSM-5) [1]. CAS core-deficit involves the planning and/or programming of the spatiotemporal parameters of movement sequences necessary for speech control [5] and is very frequently associated with an expressive language disorder [6]. 

The prevalence of CAS in the general population is low, 1–2 out of 1000 children [7], but it rises to 2.4% when considering children with SSD [8].

Despite the abovementioned differences in epidemiology and symptoms profile, an association between ASD and CAS has been suggested [9,10]. The prevalence of CAS is presumably higher in non-verbal or minimally verbal children with ASD, who represent about 25–30% of the ASD population without useful speech by age 5 [11].

ASD/CAS association is also supported by a possible shared genetic basis although only few syndromes or genes have been currently identified such as the 16p11.2 deletion syndrome [12] and the CNTNAP2 gene deletion on 7q35 position. The latter encodes a ‘neurexin’ protein that is associated with several neurodevelopmental disorders, including speech and language disorders [13] and autism [14,15]. Moreover, studies on the function of FOXP2, which may be mutated in some CAS, underscored molecular intersections between networks involved in spoken language and pathways implicated in Intellectual Disability/ASD [16]. Furthermore, genes known to be regulated by FOXP2 have been implicated in disorders such as schizophrenia (e.g., DISC1) [17] and ASD (e.g., MET and MEF2C) [18,19]. 

From a clinical perspective, both ASD and CAS children experience a delayed expressive language acquisition that might contribute to difficulties in early differential diagnosis between these two conditions. This is of outmost importance as specific and early treatments are now available for these two conditions. A further difficulty relies on the lack of specific neurobiological markers, which would help distinguishing between ASD and CAS at an early age. The use of brain MRI has increased the potential for the application of advanced techniques for detecting brain abnormalities both in ASD and CAS [20,21]. In particular, morphometric and Diffusion-Weighted Imaging (DWI) MRI studies showed an early altered brain trajectory in ASD, involving mainly the fronto-temporal and basal ganglia circuits [22,23]. Morphometric and connectivity brain MRI abnormalities have been also reported in children with CAS and other speech sound disorders, with recurrent abnormalities involving the left supramarginal gyrus, fronto-temporal regions and basal ganglia among other regions [21,24,25,26,27,28]. 

In recent years, there has been a growing interest in the identification of shared brain abnormalities across psychiatric and neurodevelopmental disorders, especially among the disorders that frequently overlap in phenotypic presentation [29]. In particular, these studies enlarge our understanding of whether symptoms’ overlap between some brain disorders could be at least partly explained by common altered neuroanatomy, or vice-versa, is subtended by disorder-specific brain underpinnings. In this framework, structural imaging studies have directly compared ASD with other neurodevelopmental disorders [30,31,32].

To our knowledge, no study has yet compared ASD and CAS through a structural morphometric MRI approach.

The main aim of this work has been to analyze structural MRI differences/similarities between children with ASD and CAS, which might share at least partly a genetic background, as well as of clinical impairment in the language domain, though in different aspects. We decided to exclude comorbid cases (ASD plus CAS) since the presence of comorbidity could be a confounding factor in the identification of disorder-specific brain underpinnings.

In particular, we aimed:

(1) To test the hypothesis that the two clinical groups (ASD and CAS) display neurostructural differences in comparison with Typically Developing children (TD) through a morphometric MRI approach (ASD vs. TD; CAS vs. TD);
(2) To investigate possible disease-specific brain structural patterns in the two clinical groups (ASD vs. CAS);
(3) To evaluate the predictive power of machine-learning analysis in differentiating these three young populations (ASD, CAS, TD).

## 2. Participants and MRI Data Acquisition

We retrospectively selected MRI brain images of patients diagnosed with ASD and CAS after a comprehensive clinical evaluation at IRCCS Stella Maris Foundation (Pisa, IT), a tertiary care university hospital. 

ASD group. Children were rigorously diagnosed with ASD according to the DSM-5 criteria [1] by a multidisciplinary team including a senior child psychiatrist, an experienced clinical child psychologist and a speech-language pathologist during three–five days of extensive evaluation. The diagnosis was confirmed by the Autism Diagnostic Observation Schedule (ADOS)-2 [33] administered by clinical psychologists who met standard requirements for research reliability. Inclusion criteria were: (a) age between 34 and 72 months, (b) Non-Verbal Intellectual Quotient (NVIQ) ≥70 and (c) spontaneous no-echolalic language of at least two-word associations, (d) absence of minimal signs potentially indicating comorbid CAS.

CAS group. CAS diagnosis was conducted by a multidisciplinary team on the basis of a comprehensive clinical, instrumental and neurological assessment as well as a video recorded speech–language evaluation. Following the international criteria for CAS diagnosis, speech and language performances were analysed by two independent expert observers according to a checklist including American Speech-Language-Hearing Association (ASHA) criteria [5] and Strand’s features of CAS [6,10,34]. Inclusion criteria were: (a) age between 34 and 71 months, (b) NVIQ ≥ 70, (c) no ASD symptoms documented by neuropsychiatric and psychological observation. 

Exclusion criteria for both clinical groups were: (a) structural anomalies detected by MRI; (b) presence of oro-facial structural abnormalities; (c) neurological or genetic diseases; (d) audiological deficits; (e) epilepsy; (f) any identified etiology of the two disorders based on DNA analysis or screening tests for inborn errors of metabolism (plasma and urine amino-acid analysis, urine organic acid measurement, urine muco-polysaccharides quantitation, plasma and urine creatine, and guanidinoacetate analysis).

A group of typically developing children (TD) who had undergone brain MRI for various reasons (including headache, seizures with fever, strabismus, cataract, paroxysmal vertigo, diplopia) was also recruited, as controls.

## 3. MRI Acquisition and Processing 

MRI data were acquired using a GE 1.5 T Signa Neuroptimized System (General Electric Medical Systems) at IRCCS Stella Maris Foundation, fitted with 40 mT/m high-speed gradients. Within the MRI protocol for children, a whole-brain fast spoiled gradient recalled acquisition in the steady-state T1- weighted series (FSPGR) was collected in the axial plane, yielding to contiguous axial slices with voxel size of 1.1 × 1.1 × 1.1 mm. All children were sedated with a general anesthesia with a halogenated agent (Sevoflurane) while spontaneously breathing. For all MRI performed between 2012 and 2018 the same sequence of acquisition was used and the written informed consent from a parent or guardian of children was obtained. This study was approved by the Pediatric Ethic Committee of Tuscany Region (Italy) through the ARIANNA Project (C52I16000020002) and RF 2016-022361560 Project, and was performed according to the Declaration of Helsinki and its later amendments or comparable ethical standards. 

We used the ARIANNA platform [35] for data handling and processing.

## 4. FreeSurfer Processing and Feature Extraction 

Structural MRI data were pre-processed according to the widely used FreeSurfer analysis pipeline (FreeSurfer v.6.0), to finally extract brain descriptive features, such as volume and thickness. FreeSurfer software is well documented and freely available for download at https://surfer.nmr.mgh.harvard.edu/. FreeSurfer is used as pre-processing workflow for structural MRI data to perform volumetric segmentation and cortical reconstruction through 31 processing steps. The technical details of these procedures are described in the publication by Fischl [36] and references therein. Briefly, this processing includes motion correction, removal of non-brain tissue using a hybrid watershed/surface deformation procedure, automated Talairach transformation, segmentation of the subcortical white matter and deep grey matter volumetric structures, intensity normalization, tessellation of the grey matter white matter boundary, automated topology correction, and surface deformation following intensity gradients to optimally place the grey/white and grey/cerebrospinal fluid borders at the location where the greatest shift in intensity defines the transition to the other tissue class. FreeSurfer is an extremely time-consuming procedure. It requires in order of a few hours (8 h on average) to segment a 3D volumetric image.

The FreeSurfer analysis pipeline computes for each structure several descriptive features. We considered in our analysis the following regional features: the volumes of subcortical structures and the volumes and thicknesses of the cortical structures, and some volume and thickness global measures. In detail, the cortex was parceled in 34 left and 34 right structures, whose volumes and thicknesses generated 138 cortical features (i.e., 68 volumes, 68 parcel thicknesses plus the mean left and mean right thickness values) contained in the *aparc* FreeSurfer output file. Thirty additional volume values were considered within those available in the *aseg* FreeSurfer output file, including the volumes of subcortical structures (thalamus, caudate, putamen, pallidum, hippocampus, amygdala, accumbens nuclei, corpus callosum and brainstem), and those of the ventricles, the subcortical cerebrospinal fluid, the optical chiasm and the cerebellum (white and gray matter). A complete list of the FreeSurfer features considered in this analysis is available in the Supplementary Materials. 

## 5. Statistical Analysis 

The entire set of brain features were statistically analysed in order to identify significant differences between patients with ASD, CAS and TD children. The ANOVA test was conducted for normally distributed features, whereas the Kruskal-Wallis test was used in case the features were not normally distributed. The Bonferroni method was used to correct the results for multiple comparisons. The effect sizes were evaluated in terms of Cohen’s *d*. Furthermore, we performed machine learning (ML)-based multivariate analysis through Support Vector Machine (SVM) binary classifiers [37]. In particular, linear-kernel SVMs were implemented to evaluate the predictive power of neuroanatomical features in the binary classification of the ASD vs. TD, CAS vs. TD, ASD vs. CAS groups. We implemented a five-fold cross-validation scheme in this analysis to partition the available data in train and test sets and to evaluate the classifier performance. The classification performance was evaluated in terms of the mean and standard deviation of area of the ROC curve (AUC) obtained across 10 repetitions of the five-fold cross-validation.

The statistical analysis and the SVM classification were carried out with Matlab R2018a (The MathWorks, Inc., Natick, Massachusetts, U.S) through in-house developed scripts and functions. In particular, the *anova1*, *kruskalwallis* and *multcompare* matlab functions were used for statistical analysis and the *fitcsvm* function for the SVM classification.

## 6. Results 

### 6.1. Participants

The initial cohort consisted of 98 patients who underwent brain structural MRI. Eighteen patients were excluded due to detection of minor brain anomalies (i.e., arachnoid cyst; periventricular leukomalacia; cortex anomalies such as heterotopia or dysplasia); seven patients were excluded due to low quality of MRI scans; five patients were excluded due to the presence of clinical comorbidities (other neurodevelopmental disorders). 

**Figure 1:**
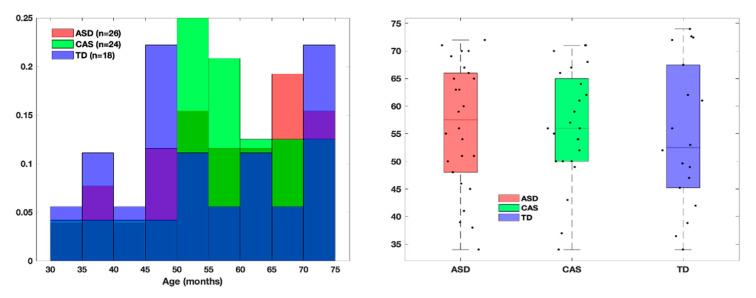
Age distribution of the subjects in the ASD, CAS and TD groups. The bar plot is shown on the left for the three groups, whereas the box plots with an overlay of the age value of the individual subjects, slightly scattered randomly along the x axis, are shown on the right. Abbreviations: ASD, Autism Spectrum Disorder; CAS, Childhood Apraxia of Speech; TD, Typical Development control.

The final cohort consisted of 68 children aged from 34 to 72 months, belonging to the three groups of ASD, CAS and TD, which were matched for gender and age. The age distributions are showed in Figure 1.

ASD group: 26 children; mean age ± standard deviation (SD) = 56 ± 11 months; CAS group: 24 children; mean age ± SD = 56 ± 10 months. TD group: 18 children; mean age ± SD = 55 ± 13 months. 

Children’s demographic characteristics are shown in Table 1 and Figure 1.

### 6.2. Statistical Analysis

**Figure 2:**
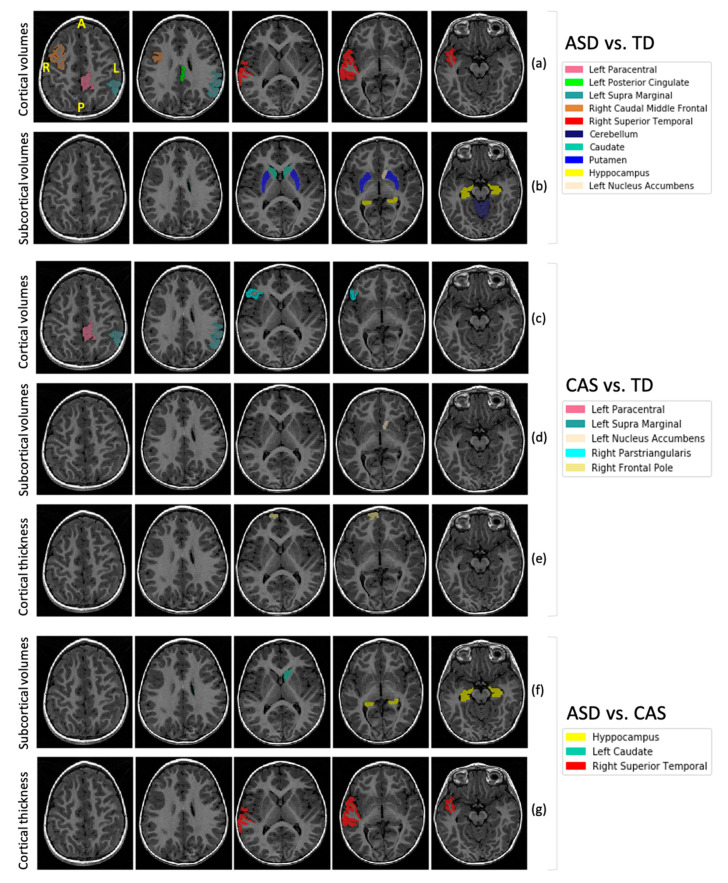
Visualization of brain regions whose features showed statistically significant differences among the three groups of subjects (see Table 2 for the complete list of features, including also global volumes). The overlay of FreeSurfer segmented regions onto the anatomical image of a representative subject is shown for specific alterations in the ASD vs. TD (**a**,**b**), CAS vs. TD (**c**–**e**) and ASD vs. CAS (**f**,**g**) comparisons. The involved set of features, i.e., the cortical and subcortical volumes, and the cortical thicknesses, are indicated in each figure row.

Statistical analysis of the complete set of FreeSurfer features was carried out. Significant differences in several brain structures across the three cohorts were detected through ANOVA statistical analysis and the Kruskall-Wallis test, as reported in Table 2 for cortical volumes, cortical thicknesses and subcortical volumes. The effect sizes in terms of Cohen’s *d* are also reported in the table. A visual representation of all altered brain regions, as segmented by FreeSurfer, is shown in Figure 2, where their overlay on the anatomical image of a representative subject is reported. In particular, brain regions whose features showed statistically significant differences in the comparison among the three groups of children are highlighted. We summarize in the text below the results of the between-group comparisons ASD vs. TD, CAS vs. TD and ASD vs. CAS, reported in Table 2 column (a), Table 2 columns (b) and (c), and Table 2 column (d), respectively. 

### 6.3. Comparison between ASD and TD

The significant neuroanatomical differences between ASD and TD are reported in Table 2 (a), visualized in Figure 2a,b and discussed below. 

Cortical volumes. Overall, ASD presented increased volumes in comparison with TD. No contra-comparison results were found. The Cohen’s *d* effect sizes vary from medium effect size to large effect size. ASD presented increased cortical volumes within the fronto-temporal lobe: left paracentral volume (*d* = 0.83), left posterior cingulate volume (*d* = 0.73), left supramarginal volume (*d* = 0.58), right caudal middle frontal volume (*d* = 0.77) and right superior temporal volume (*d* = 0.95).

Subcortical volumes. Overall, ASD presented increased subcortical volumes in comparison with TD; no contra comparison results were found. ASD presented increased volumes in the following regions: caudate (left *d* = 1 and right *d* = 0.89), putamen (left *d* = 0.89 and right *d* = 0.88), hippocampus (left *d* = 1.15 and right *d* = 1.19) and left nucleus accumbens (*d* = 0.92).

Cortical thickness. No statistically significant results were detected.

Global measures and cerebellum. An increase in cortex volumes of the cerebellum (left *d* = 0.97 and right *d* = 1) was detected in children with ASD with respect to TD. Children with ASD also presented an increased global subcortical grey matter volume (*d* = 0.97) and total grey matter volume (*d* = 0.71) with respect to TD.

### 6.4. Comparison between CAS and TD

The significant neuroanatomical differences between CAS and TD are reported in Table 2 (b) and (c), visualized in Figure 2c–e, and discussed below.

Cortical volumes. Overall, children with CAS presented higher values of cortical volume within the frontal lobe, in particular in left paracentral (*d* = 0.80), right pars triangularis (*d* = 0.52) and in left supramarginal (*d* = 0.50).

Subcortical volumes. CAS showed an increased volume in left nucleus accumbens (*d* = 0.97) with respect to TDs.

Cortical thickness. CAS presented reduced values of cortical thickness in the frontal lobe, in particular in the right frontal pole (*d* = 0.97). 

Global measures and cerebellum. No statistically significant results were detected.

### 6.5. Comparison between ASD and CAS

The significant neuroanatomical differences between ASD and CAS are reported in Table 2 (d), visualized in Figure 2f,g, and discussed below. 

Cortical volumes. No statistically significant differences between ASD and CAS were directly detected in cortical volumes.

Subcortical volumes. Statistically significant greater volumes of the left caudate (*d* = 0.68) and of the hippocampi (left *d* = 0.57 and right *d* = 0.56) have been detected in ASD with respect to CAS.

Cortical thickness. ASD showed statistically significant higher cortical thickness in the right superior temporal (*d* = 0.89) in comparison to CAS.

Global measures and cerebellum. No statistically significant results were detected.

### 6.6. Machine Learning Analysis

The performances obtained with the binary linear-kernel SVM in the ASD vs. TD, CAS vs. TD, ASD vs. CAS classification are reported in Table 3. The classification performance achieved by different groups of features (e.g., cortical volumes/thickness, subcortical volumes, and their combinations) are reported in the table in terms of the ROC curve (AUC) values obtained according to a five-fold cross-validation scheme. The error assigned to each AUC value is computed as the standard deviation over 10 repetitions of the five-fold cross-validation.

It can be noticed from Table 3 that the most informative groups of features driving the discrimination performance between children with ASD and TD are the subcortical volumes, either including or not the global measures. In those cases, AUC values of 0.76 ± 0.14 and of 0.75 ± 0.16 have been obtained. The classification performance remains high when the combination of all features is considered, leading to an AUC of 0.73 ± 0.19. 

By contrast, the group of children with CAS does not appear to be distinguishable from the group of TD children by means of a linear-kernel SVM classification. Despite that the classification performance obtained is not above the chance level, the set of cortical thickness features showed an average AUC value of 0.62, and the combination of all features an AUC of 0.61. 

It turns out also that the groups of children with ASD and with CAS are indistinguishable from each other by means of a linear-kernel SVM classification, as reported in the rightmost column of Table 3 (all chance-level AUC values). In this case, only the set of cortical thickness features displayed an average AUC value of about 0.64. 

## 7. Discussion 

To our knowledge, this is the first structural morphometric MRI study comparing ASD, CAS and TD. This may represent the first step towards understanding the neural substrate that characterizes the two conditions, possibly being of utmost importance for future early tailored intervention strategies. Furthermore, it could be helpful to understand as early as possible whether children with non-verbal or minimally verbal ASD have a CAS on the basis of neuroanatomical brain configuration, given the difficulty of directly testing these children. 

### 7.1. Are ASD and CAS Brain Different from TD Brain?

#### 7.1.1. ASD Versus TD

ASD children displayed an overall increase of total grey matter volume in comparison with TD, specifically distributed in the fronto-temporal regions. 

Grey matter volume increase is one of the most consistent structural findings in ASD, and it is particularly striking in younger children [20], thus supporting the early brain overgrowth of ASD related to abnormal cortical development and expansion [38,39]. It is of interest that the cortical volumes’ increase found in our ASD children versus TD is mainly distributed in fronto-temporal lobes, known to be crucial for socio-communicative skills development [40]. The importance of these two brain lobes in the pathophysiology of ASD was further corroborated by two recent studies [41,42], which identified both frontal and temporal lobe volumes as the most discriminative features in the ASD-control classification. Critically, the left supramarginal gyrus, which belongs to the inferior parietal lobule, was also increased in volume in ASD children. This region is massively connected with both Broca’s and Wernicke’s areas, and it is altered during processing of some language aspects, including pragmatics [43], and sentence comprehension [44] in children with ASD.

Besides the well-replicated alterations in the fronto-temporal cortical regions, an increased volume in some subcortical structures was detected in the current work. Specifically, both right and left hippocampi were significantly increased in ASD group when compared with TD. The hippocampus is considered relevant for the ASD pathophysiology, since it is connected to the amygdala within the limbic system and implicated in crucial functions of the “social brain” [45]. Our result of increased hippocampi volumes is consistent with findings from a large study of 98 patients with ASD (age-range: 7.5–18 years) compared to 31 controls [46], and it is also concordant with a longitudinal investigation in children between the ages of 8 and 12 years [47]. However, findings on hippocampal volume in autism are quite controversial, as some authors did not identify differences compared with controls [48], while significantly decreased volumes were found by other authors [49,50].

In addition, we identified a significant enlargement of the striatum (the part of the basal ganglia composed of three subnuclei: caudate, putamen, and nucleus accumbens) in patients with ASD in comparison with TD. Structural alterations of the basal ganglia, and in particular of the caudate nucleus and the putamen, have been frequently detected in ASD, both in children [51] and adults [52], and associated with the severity of restricted and repetitive behaviors [53]. Instead, the nucleus accumbens is generally reported as a critical node within the brain’s reward circuitry, but, more broadly, it is also involved in action selection, integration of cognitive and affective information, and suppression of inappropriate actions [54].

Interestingly, decreased structural and functional connectivity between the ventral tegmental area of the midbrain and the nucleus accumbens, namely two brain areas crucial for processing social reward, was found in ASD children compared with TD children [55]. Such brain alterations appear to be related to the level of social interaction impairments in patients with ASD, providing support for the link between abnormalities in reward processing and widespread deficits in social engagement and communication in autism (i.e., the social motivation theory [56]).

The increased cerebellum volume we found is in agreement with some [49,57], but not all [20,58] literature findings. The cerebellum has been traditionally considered to be primarily involved in motor control and coordination, but its function comprises other domains typically impaired in ASD children, such as language, social cognition, and affective regulation [59]. In keeping with these data, a consensus paper recently highlighted a crucial role of the neuroanatomical cerebellar alterations in ASD [60].

We also detected an increased volume in the right superior temporal gyrus (STG), a region implicated in the processing of semantic [61] and prosodic cues [62], thus being two language aspects frequently impaired in children with ASD [63]. In this context, two previous fMRI investigations observed increased brain activation in the right STG of ASD children during processing of prosodic cues, such as anger and irony [64,65], that may reflect a more effortful processing required for the interpretation of prosodic information. The increased grey matter volume in right STG detected in the current study is consistent with three previous sMRI investigations focused respectively on toddlers (mean age: 30 months) [66], preschoolers (mean age: 53 months) [67], and children/adolescents (age range: 8.8–18.3 years) [68] with ASD, supporting the view that volumetric alterations in this region are present across the developmental age.

Cortical thickness analysis reported, in the current sample, no significant differences between ASD patients and TD peers. Some previous studies have shown cortical thickness differences between participants with ASD and controls across the whole brain, and in particular, a greater cortical thickness in fronto-temporal regions implicated in the processing of language and social information [69,70,71]. Nevertheless, other investigations have failed to detect between-groups differences in cortical thickness [72]. Additionally, cortical thinning in the pars opercularis of the inferior frontal gyrus was reported both in preschoolers [67] and in adults [73] with ASD. These discrepancies could be partly explained by study differences in sample size, age, patients’ characteristics and clinical symptoms’ severity, MRI acquisition and processing protocols. Longitudinal studies identified that differences in cortical thickness between patients with ASD and TD controls change over time [74,75], suggesting that an altered cortical thickness trajectory may constitute a more reliable neuroanatomical marker in ASD.

#### 7.1.2. CAS Versus TD

Apraxia of speech (AOS) is the main symptom in adults after infarcts to the left hemisphere involving the inferior frontal region, in particular the posterior part of Broca’s area [76] and the insular cortex [77] or adjacent white matter [78]. Instead, Childhood Apraxia of Speech (CAS) is a developmental disorder whose brain correlates remain largely unknown and little evidence is available to date [21,24,28].

Our results show structural brain differences in children with CAS in comparison with TD. In particular, altered cortical volumes in areas crucial for speech and language were found, with increased volumes distributed within the parietal lobe (supramarginal gyrus), the frontal lobe (para-central, pars triangularis), and decreased volume in the nucleus accumbens. 

The involvement of the left supramarginal gyrus in CAS has been previously described by Kadis [24], who found an increased thickness of this region in children with CAS aged as in our sample. Anatomo-clinical correlation studies in brain-damaged patients with a selective impairment of the auditory-verbal memory span indicate that the inferior parietal lobule (supramarginal gyrus) of the left hemisphere, at the temporo-parietal junction, represents the main neural correlate of the ‘store’ component of phonological short-term memory [79]. The left supramarginal gyrus is reported to play an important role in speech production, its damage thus being associated with deficits in phonemic discrimination and speech planning [80]. Furthermore, the supramarginal gyrus is part of a neural dorsal pathway receiving inputs from the auditory cortex and has reciprocal connections with the opercular part of the inferior frontal gyrus and the ventral premotor area [81,82], both involved in articulatory planning [83]. Indeed, as recently suggested by Nakamichi et al. [84] in a functional Near Infrared Spectroscopy (fNIRS) study, the supramarginal gyrus seems to be involved in phonemic processing and articulatory learning through an “articulatory loop” in which phonemic and oral somatosensory information are mapped onto motor representations for articulation. Partially overlapping with our findings, morphological abnormalities of supramarginal gyrus and, bilaterally, of temporal planum and Heschl’s gyrus have been described in children with a subtype of speech sound disorder characterized by persistent speech sound errors [27]. 

Another resulting altered region in terms of increased values in CAS vs. TD in the current work was the pars triangularis within the Broca’s area [85,86,87]. This region has been associated with linguistic processes, including syntax and semantics [86,88,89], but its precise functional role still remains controversial. Recently, Elmer and colleagues [90] suggested that pars triangularis can be considered as a “hub” region of the language-control network and would have a role in supporting verbal working memory functions during simultaneous language translation. 

A further area within the frontal lobe founded as volumetrically increased in CAS vs. TD was the left paracentral region, a sensory-motor area involved in motor control in adults, whose eventual role in the acquisition of speech motor control during development is still unknown. 

However it is of note that the paracentral region is contiguous to the precentral region reported as altered in the diffusion study on children with CAS by Fiori et al. [21].

At subcortical level, the nucleus accumbens, which is part of the ventral striatum and belongs to a broad language learning network [91], showed a decreased volume in CAS compared to controls.

As far as cortical thickness is concerned, the right frontal pole showed reduced values in CAS compared to TD. It is of note that the frontal pole (Broadmann Area 10) has been described as the most evolved region in humans [92] and it is essential for attention control, manipulation of stored knowledge and modulation of complex actions, cognition emotion, and behaviour [93]. However, the interpretation of this finding in children with CAS is still unclear.

### 7.2. Which Regions Directly Differentiate ASD vs. CAS?

Comparison between ASD and CAS showed that the caudate and the hippocampus volumes, together with the superior temporal thickness, were shown to be increased in ASD vs. CAS. 

The basal ganglia (BG), which consist of the striatum, caudate nucleus and putamen, are involved in motor function [94] as well as in learning and memory processes [95] and have been widely related to the repetitive and stereotyped behaviors characteristic of autism spectrum [96,97]. Bilaterally reduced grey matter density in the caudate nuclei was described in patients with CAS related to FOXP2 mutations. In particular, MRI studies of the Ke family [98] and a recent report of an unrelated male child with a FOXP2 intragenic deletion [99] confirmed reductions of the caudate nucleus bilaterally, as well as of the globus pallidus and hippocampus. 

As above mentioned in the ASD vs. TD section, the superior temporal gyrus has been widely described as having a crucial role in the social brain development, and not surprisingly has been found to be increased in our ASD population. Incidentally, the superior temporal gyrus is also involved in language comprehension that is often more severely impaired than language production in children with ASD [100], whereas an inverse profile characterizes children with CAS.

Data concerning hippocampus increased volumes in ASD are consistent with the findings of other studies, whereas there is only a case report by Liéogeois et al. (2016) [99] describing bilateral hippocampal and basal ganglia volume reduction (thalamus, globus pallidus, and caudate nucleus) in a 8 years old child with FOXP2 related CAS 

Since the abovementioned regions (caudate, superior temporal gyrus and hippocampus) significantly differentiated not only ASD from CAS, but also ASD from TD, it may be hypothesized that these results are more ascribable to ASD higher values than to CAS lower values in the direct comparison. 

### 7.3. Is Machine Learning Informative about Diagnosis Prediction?

Non-invasive brain imaging techniques coupled with advanced image analysis methodologies based on machine learning (ML) have been recently used to provide an automated classification of diseases, including ASD [101], whereas no application in the CAS field is yet reported. We have included, then, a ML analysis in the current work to estimate the predictive capabilities of neuroanatomical descriptive features in a binary comparison between two out of the three groups of children.

In the cases of CAS vs. TD and of ASD vs. CAS comparisons, the performances achieved did not score above the chance level. This means that the two groups are quite indistinguishable at least with linear-kernel SVM. It has to be noted that the high standard deviation assigned to the AUC values is mainly due to the limited sample size, which did not allow avoidance of the overfitting problem. Increasing the dataset population could sensibly reduce the standard deviation on the AUC values and the classification trends that are barely visible (AUC~0.6) may become apparent. It is also worth mentioning that any subtle relationship between neuroanatomical features that may characterize the two clinical groups of this study could be very hard to catch by a linear classifier. Non-linear approaches could be implemented. However, even in this case, the limited data samples (less than 30 subjects per group) with respect to the large amount of image features (sets from ~30 to more than 150 features have been considered) would not avoid the occurrence of overfitting, which causes a reduction in the generalization ability of the classifier.

In conclusion our ML analysis highlights that the group of children with ASD shows a distinct brain pattern with respect to the control group, focused especially on subcortical brain regions. The comparison between the children with CAS and the control group showed a trend towards the possibility of identifying a relevant pattern focused on cortical thickness, that represents the only relevant trend in the direct comparison between the two clinical groups. Indeed, while ASD present more widespread brain alterations, confirming previous data and opening the possibility to predictive power of ML algorithms based on MRI data, a predictive role of brain patterns in CAS is still not supported by the current sample size.

### 7.4. Final Considerations 

In summary our work reports a general consistency with the previous literature in the brain structural comparison between ASD versus TD and CAS versus TD, with resulting effect size ranging from medium to large. It is of interest that all but one structure showed higher volumes in both ASD and CAS versus TD in this young population, in accordance with previous literature, especially for the ASD group. The only opposite trend regards cortical thickness (CAS < TD in frontal pole cortical thickness), but the significance of this result is not clear, cortical thickness not being univocally interpreted in the literature, and being age-dependent [102]

The more widespread abnormalities have been observed comparing ASD vs. TD, while more focused alterations have been found by comparing CAS vs. TD. Considering all the structures (cortical and subcortical volumes and cortical thickness), ASD alterations involve fronto-temporal regions together with basal ganglia and cerebellum, while CAS alterations seem to be more focused on and shifted to frontal regions, thus suggesting a possibly more specific speech-language related distribution of anomalies. We can speculate that autistic children’s brain atypicalities are more widespread in the superior temporal gyrus, historically considered the site of sound processing and auditory association cortex, but also linked to social cognition and implicated in visual analysis of social information. Moreover, the superior temporal gyrus has been involved in the perception of the emotional facial stimuli and more generally in social interactions. Conversely, atypicalities in CAS are more shifted in the frontal regions, where sensory motor circuitries are represented. Furthermore, it can be considered that overlapping structural regions between the two conditions can assume a different role at a functional level. 

### 7.5. Strenghts and Weaknesses of the Study

To the best of our knowledge this is the first study comparing young children presenting with ASD, CAS and TD at a neurostructural level. Though our results suggest the possibility of detecting brain correlates potentially able to disentangle the two conditions from typical development from an early age (when clinical specific phenotypization is not easy), ML analysis in our sample has partially reached an optimal predictive power. Indeed, while our ML analyses confirm the possibility of detecting brain patterns with reliable predictive power in the ASD vs. TD comparison, this is not the same for CAS vs. TD and ASD vs. CAS. This can be ascribable to limited sample size, which does not allow a complete representation of the different distribution of possible alterations in the two conditions and also prevents the possibility of implementing more complex non-linear classifier models.

Certainly, the study has strengths and weakness. First, this is a whole brain analysis not driven by an a priori hypothesis, thus minimizing possible interpretation biases. Second, clinical groups, with a short age range, have been evaluated from expert clinicians (multidisciplinary team) in the field of ASD and CAS. Thirdly, the single-site recruitment of study participants limited the noise associated with the collection of data from different MRI scanners and different sequences. Our results should be interpreted in the light of some methodological limitations among which the small sample size is the most crucial. Indeed, our sample size of about 20 children per group is quite small, thus limiting the generalizability of the results and the strength of the conclusions [103]. Hence, it will be critical in the future to recruit larger samples in order to replicate our findings, and to provide a more robust characterization of the clinical and neuroanatomical profiles of children with ASD and CAS. This study’s limits are mitigated by the fact that patients have been carefully selected so as to be included in a limited age-range, in order to minimize age dependent structural brain alterations. Notably, the sample has been reduced from the initial cohort with the aim of obtaining a well-selected and homogeneous pool of MRI data, coupled with an extensive multidisciplinary clinical characterization of patients.

Furthermore, the retrospective nature of this study has not allowed us to apply appropriate clinical evaluations, such as ADOS-2 to the CAS population and standardized CAS protocols to the ASD population. 

Future studies should collect a further group of patients with ASD and comorbid CAS in order to investigate the extent to which these cases differ from the “pure” disorders.

Though remaining cautious about interpretation, our results may represent the first step towards understanding the neural substrate that characterizes ASD and CAS conditions and therefore, in the future, for identifying neurobiological markers that may support early diagnosis of non-verbal or minimally verbal children with ASD.

Furthermore, the identification of a specific motor speech disorder associated to ASD is crucial for tailoring an appropriate early intervention. 

## Figures and Tables

**Table 1:** Demographic characteristics of the cohorts of subjects with ASD, with CAS and TD subjects. The number of subjects in each subgroup is reported (*n*) together with the male/female percentage with respect to the total number of subjects in each cohort.

Age in Months (Mean ± std [Range]) by Subjects’ Category					
ASD (*n* = 26)		CAS (*n* = 24)		TD (*n* = 18)	
56 ± 11 (34–72)		57 ± 10 (34–71)		55 ± 13 (34–74)	
Males	Females	Males	Females	Males	Females
(*n* = 20, 77%)	(*n* = 6, 23%)	(*n* = 18, 75%)	(*n* = 6, 25%)	(*n* = 13, 72%)	(*n* = 5, 28%)
57 ± 11 [34,35,36,37,38,39,40,41,42,43,44,45,46,47,48,49,50,51,52,53,54,55,56,57,58,59,60,61,62,63,64,65,66,67,68,69,70,71]	54 ± 12 [39,40,41,42,43,44,45,46,47,48,49,50,51,52,53,54,55,56,57,58,59,60,61,62,63,64,65,66,67,68,69,70,71,72]	56 ± 10 [34,35,36,37,38,39,40,41,42,43,44,45,46,47,48,49,50,51,52,53,54,55,56,57,58,59,60,61,62,63,64,65,66,67,68,69,70,71]	57 ± 12 [37,38,39,40,41,42,43,44,45,46,47,48,49,50,51,52,53,54,55,56,57,58,59,60,61,62,63,64,65,66,67,68]	58 ± 12 [39,40,41,42,43,44,45,46,47,48,49,50,51,52,53,54,55,56,57,58,59,60,61,62,63,64,65,66,67,68,69,70,71,72,73,74]	47 ± 13 [34,35,36,37,38,39,40,41,42,43,44,45,46,47,48,49,50,51,52,53,54,55,56,57,58,59,60,61,62,63,64,65,66,67]

Abbreviations: ASD, Autism Spectrum Disorder; CAS, Childhood Apraxia of Speech; TD, Typical Development control.

**Table 2:** Cortical volumes showing a statistically significant difference in the comparison among children with ASD, children with CAS and TD children. The test statistics (either ANOVA or Kruskal-Wallis), the p-values, the specification of which group comparisons showed statistically significant differences and the related effect size in terms of Cohen’s *d* are reported.

Comparison among ASD, CAS and TD Groups		Statistical Test ^§^		Cohen’s *d* in the Between-Group Comparisons			
				(a)	(b)	(c)	(d)
		F/X^2^	*p* Value	ASD > TD	CAS > TD	CAS < TD	ASD > CAS
**Cortical volumes**	Left Paracentral volume	4.1	0.02	0.83	0.79	/	/
	Left Posterior Cingulate volume	4.0	0.02	0.73	/	/	/
	Left Supra Marginal volume ^§^	7.7	0.02	0.58	0.48	/	/
	Right Caudal Middle Frontal volume ^§^	5.9	0.05	0.77	/	/	/
	Right Pars Triangularis volume ^§^	8.3	0.01	/	0.53	/	/
	Right Superior Temporal volume	5.9	0.004	0.95	/	/	/
**Cortical Thickness**	Right Superior Temporal thickness	4.1	0.02	/	/	/	0.79
	Right Frontal Pole thickness	4.1	0.02	/	/	0.97	/
**Subcortical structures, cerebellum and global measures**	Left Caudate volume	5.8	0.005	1.04	/	/	0.68
	Left Cerebellum Cortex volume	4.1	0.02	0.97	/	/	/
	Left Hippocampus volume ^§^	12	0.002	1.15	/	/	0.57
	Left Nucleus Accumbens ^§^	11	0.004	0.92	0.97	/	/
	Left Putamen volume ^§^	7.7	0.02	0.89	/	/	/
	Right Caudate volume ^§^	8.0	0.02	0.89	/	/	/
	Right Cerebellum Cortex volume	4.5	0.01	1.02	/	/	/
	Right Hippocampus volume ^§^	12	0.002	1.19	/	/	0.56
	Right Putamen volume ^§^	9.3	0.01	0.88	/	/	/
	SubCortical Gray matter volume	5.3	0.008	0.97	/	/	/
	Total Gray matter volume	3.1	0.05	0.71	/	/	/

Abbreviations. ASD, Autism Spectrum Disorder; CAS, Childhood Apraxia of Speech; TD, Typical Development control. ^§^ Features with not-normal distribution undergone Kruskal-Wallis test instead of ANOVA.

**Table 3:** Classification performance in the binary classification between the ASD vs. TD, CAS vs. TD and ASD vs. CAS groups using structural features. The performances are expressed in terms of the area under the ROC curve (AUC) achieved in the classification of different groups of features (e.g., cortical volumes/thickness, subcortical volumes, global volumes and their combinations). The cross-validation scheme implemented consisted in 10 repetitions of a five-fold cross validation.

Features		AUC (Mean ± SD)		
		ASD vs. TD	CAS vs. TD	ASD vs. CAS
		(*n* = 44)	(*n* = 42)	(*n* = 50)
Subcortical volumes + cerebellum	m = 34	0.75 ± 0.16	0.48 ± 0.17	0.42 ± 0.14
Subcortical volumes and global measures	m = 38	0.76 ± 0.14	0.54 ± 0.18	0.45 ± 0.12
Cortical volumes	m = 68	0.53 ± 0.17	0.52 ± 0.18	0.45 ± 0.17
Cortical thicknesses	m = 70	0.52 ± 0.19	0.62 ± 0.21	0.64 ± 0.17
All cortical features (volumes and thicknesses)	m = 138	0.63 ± 0.18	0.59 ± 0.17	0.50 ± 0.15
All structural features and global measures	m = 176	0.73 ± 0.19	0.61 ± 0.17	0.45 ± 0.16

Abbreviations: AUC, area under the ROC curve; SD, standard deviation; m, number of features in each selected group of features (each table row); *n*, number of subjects used in each classification problem (each table column); ASD, Autism Spectrum Disorder; CAS, Childhood Apraxia of Speech; TD, Typical Development control.

## Supplementary Materials

The following are available online at https://www.mdpi.com/2075-4426/10/4/275/s1, Table S1: FreeSurfer features considered in the analysis.
