# Supplementary material for: Autism Spectrum Disorder and Childhood Apraxia of Speech: Early Language-Related Hallmarks across Structural MRI Study
Source: J Pers Med. 2020 Dec 12;10(4):275. doi: 10.3390/jpm10040275 (PMC7768516; doi:10.3390/jpm10040275)
Supplement: Supplementary file 1 [file jpm-10-00275-s001.pdf]

# Autism Spectrum Disorder and Childhood Apraxia of speech: early language-related hallmarks across structural MRI study

Eugenia Conti[1], Alessandra Retico[3], Letizia Palumbo[3], Giovanna Spera[3], Paolo Bosco[1], Laura Biagi[1], Simona Fiori[1], Michela Tosetti[1], Paola Cipriani[1], Giovanni Cioni[1,2], Filippo Muratori[1,2], Anna Chilosi[1*], Sara Calderoni[1,2*]

[1]*IRCCS Fondazione Stella Maris, Pisa, Italy,*

[2]*Department of Clinical and Experimental Medicine, University of Pisa, Pisa, Italy,*

[3]*INFN, Pisa Division, Pisa, Italy*

## Supplementary Material

### S1. FreeSurfer analysis of brain MRI data

The FreeSurfer (FS, v.6.0) analysis pipeline (https://surfer.nmr.mgh.harvard.edu/) [1][2], has been implemented to carry out the segmentation of brain MRI in substructures and to compute a set of neuroanatomical descriptive features for each subject.
Firstly, we have converted 3D T1-weighted MR brain images from DICOM format to Nifti format.
Secondly, we have used the FS pre-processing workflow, known as *recon-all* analysis pipeline, which runs the input structural MRI scan across several FS functions performing all cortical reconstruction through 31 processing steps. The *recon-all* syntax is: *recon-all -i <input MRI scan> -s <subject id that you make up> -sd <directory to put the subject folder in> -all*
Lastly, we have extracted the volumes ($mm^3$) and thickness ($mm^2$) from the *aseg.stats* and *h.aparc.stats recon-all* output files, using *asegstats2table* and *aparcstats2table* FS scripts. These software generate tables of segmented subcortical volume and cortical parcellation statistics for each structure. The execution of this workflow is made automated for all MRI scans using a bash script.
A complete list of the FreeSurfer features considered in this analysis is provided in table S1.

### S1. Statistical analysis

The statistical analysis of brain features carried out to identify significant differences between patients with ASD, CAS and TD children has been conducted according to the ANOVA test [3] for normally distributed features, whereas the Kruskal-Wallis [4] test in case the features were not normally distributed. The normality was evaluated by the Shapiro-Wilk test [5]. The statistical analysis was carried out with Matlab R2018a (The MathWorks, Inc.) through in-house built scripts and functions exploiting the *anova1, kruskalwallis* and *multcompare* matlab functions.
The Bonferroni method was used to correct the results for multiple comparisons [6]. The effect sizes were evaluated in terms of the Cohen's d [7].
To evaluate whether neuroanatomical characteristics are predictive either of the ASD or the CAS conditions, we performed a machine learning (ML)-based multivariate analysis. We implemented Support Vector Machine (SVM) binary classifiers [8] through the Matlab R2018a function *fitcsvm*. In particular, linear-kernel SVM were implemented to evaluate the predictive power of neuroanatomical features in the binary classification of the ASD vs. TD, CAS vs. TD, ASD vs. CAS groups. We implemented a 5-fold cross-validation scheme [9] in this analysis to partition the available data in train and test sets and to evaluate the classifier performance. The classification performance was evaluated in terms of the mean and standard deviation of area of the ROC curve (AUC) [10].

Table S1. **FreeSurfer features considered in the analysis**. Cortical volumes of 34 structures of the left (L) and right (R) hemispheres; cortical thickness of 34 structures of the L and R hemispheres and the average cortical thickness; 12 volumes of either subcortical or cerebellar L and R structures; volumes of 16 other structures, including white matter structures, brainstem and global measures.

| Cortical volumes (L and R) | Cortical thickness (L and R) | Subcortical and cerebellum volumes (L and R) |
|---|---|---|
| Bankssts volume | Bankssts thickness | Lateral ventricle volume |
| Caudal anterior cingulate volume | Caudal anterior cingulate thickness | Inferior lateral ventricle volume |
| Caudal middle frontal volume | Caudal middle frontal thickness | Cerebellum white matter volume |
| Cuneus volume | Cuneus thickness | Cerebellum cortex volume |
| Entorhinal volume | Entorhinal thickness | Thalamus proper volume |
| Fusiform (gyrus) volume | Fusiform (gyrus) thickness | Caudate volume |
| Inferior parietal volume | Inferior parietal thickness | Putamen volume |
| Inferior temporal volume | Inferior temporal thickness | Pallidum volume |
| Isthmus cingulate volume | Isthmus cingulate thickness | Hippocampal volume |
| Lateral occipital volume | Lateral occipital thickness | Amygdala volume |
| Lateral orbitofrontal volume | Lateral orbitofrontal thickness | Nucleus Accumbens Area volume |
| Lingual volume | Lingual thickness | Ventral DC (Diencephalon) volume |
| Medial orbital frontal volume | Medial orbital frontal thickness | |
| Middle temporal volume | Middle temporal thickness | **Other subcortical volumes and global measures** |
| Parahippocampal volume | Parahippocampal thickness | Third Ventricle volume |
| Paracentral volume | Paracentral thickness | Fourth Ventricle volume |
| Pars opercularis volume | Pars opercularis thickness | Brain stem volume |
| Pars orbitalis volume | Pars orbitalis thickness | Cerebrospinal fluid volume |
| Pars triangularis volume | Pars triangularis thickness | Optic chiasm volume |
| Pericalcarine volume | Pericalcarine thickness | Posterior cingulate cortex volume |
| Postcentral volume | Postcentral thickness | Middle posterior cingulate cortex volume |
| Posterior cingulate volume | Posterior cingulate thickness | Central cortex volume |
| Precentral volume | Precentral thickness | Middle anterior cingulate cortex volume |
| Precuneus volume | Precuneus thickness | Anterior cingulate cortex volume |
| Rostral anterior cingulate volume | Rostral anterior cingulate thickness | Cortex volume |
| Rostral middle frontal volume | Rostral middle frontal thickness | Cerebral white matter volume |
| Superior frontal volume | Superior frontal thickness | Subcortical gray matter volume |
| Superior parietal volume | Superior parietal thickness | Total gray matter volume |
| Superior temporal volume | Superior temporal thickness | Brain segmented volume without ventricles (BrainSegVolNotVent) |
| Supra-marginal volume | Supra-marginal thickness | Estimated Total Intracranial Volume (eTIV) |
| Frontal pole volume | Frontal pole thickness | |
| Temporal pole volume | Temporal pole thickness | |
| Transverse temporal pole volume | Transverse temporal pole thickness | |
| Insula volume | Insula thickness | |
| | Cortical thickness mean | |

## References


[1] Fischl B. FreeSurfer. Neuroimage 2012. https://doi.org/10.1016/j.neuroimage. 2012.01.021

[2] Klein A, Tourville J. 101 labeled brain images and a consistent human cortical labeling protocol. Front Neurosci 2012;6:1–12. https://doi.org/10.3389/fnins.2012.00171.

[3] Fisher R. "The Correlation between Relatives on the Supposition of Mendelian Inheritance." Transactions of the Royal Society of Edinburgh, 52(2), 1918. Pages 399-433.
[4] William H. Kruskal and W. Allen Wallis. Use of ranks in one-criterion variance analysis. Journal of the American Statistical Association 47 (260): 583–621, December 1952.
[5] Sam S. Shapiro, Martin Bradbury Wilk (1965). "An analysis of variance test for normality (complete samples)", Biometrika, 52, 3 e 4, pagine 591-611.
[6] McDonald, J. H. (2009). Handbook of biological statistics (Vol. 2, pp. 6-59). Baltimore, MD: sparky house publishing.
[7] Cohen, J. (1988). Statistical power analysis for the behavioral sciences, 2nd ed. Lawrence Erlbaum Associates, Publishers
[8] Vapnik, VN NV. The nature of statistical learning theory. Berlin, Heidelberg: Springer-Verlag (1995).
[9] Stone, M (1974). "Cross-Validatory Choice and Assessment of Statistical Predictions". Journal of the Royal Statistical Society: Series B (Methodological). 36 (2): 111–147.
[10] Metz CE. Receiver operating characteristic analysis: a tool for the quantitative evaluation of observer performance and imaging systems. J Am Coll Radiol (2006) 3:413–22.
